# Associations of postural asymmetry with refractive error: an objective photogrammetric analysis in students

**DOI:** 10.3389/fmed.2026.1727688

**Published:** 2026-01-29

**Authors:** Xuan Li, Qingwen Yang, Ziyan Ma, Jianhua Huang, Yang Jian, Tianyu Chen, Haoyan Wang, Dongfeng Li, Juan Du, Ke Wu, Huan Liu, Yu Cao, Zhengzheng Wu, Bolin Deng

**Affiliations:** 1 Department of Ophthalmology, Sichuan Provincial People’s Hospital, Chengdu, Sichuan, China; 2 School of Medicine, University of Electronic Science and Technology of China, Chengdu, Sichuan, China; 3 Genetic Diseases Key Laboratory of Sichuan Province, Department of Laboratory Medicine, Sichuan Academy of Medical Sciences and Sichuan Provincial People’s Hospital, University of Electronic Science and Technology of China, Chengdu, Sichuan, China; 4 Research Unit for Blindness Prevention of Chinese Academy of Medical Sciences (2019RU026), Sichuan Academy of Medical Sciences and Sichuan Provincial People’s Hospital, Chengdu, Sichuan, China; 5 Medical School of Ophthalmology & Optometry, North Sichuan Medical College, Nanchong, Sichuan, China; 6 Health Profession, University of the West of England, Bristol, United Kingdom

**Keywords:** adolescents, anisometropia, axial length, myopia, postural misalignment

## Abstract

**Purpose:**

To assess associations between objectively measured postural misalignments and refractive errors in Chinese adolescents.

**Methods:**

A school-based, cross-sectional study was conducted on 567 students aged 12–18 years. Participants underwent comprehensive eye examinations including cycloplegic autorefraction and axial length (AL) measurement and objective postural quantification using a photogrammetric system. Questionnaire-derived covariates for myopia risk factors were available for 258 participants. Multivariable logistic and linear regression models assessed associations between postural misalignments and refractive outcomes.

**Results:**

The study population (mean age: 14.96 ± 1.60 years; 43.4% male) exhibited a high prevalence of myopia (82.5%) and anisometropia (34.7%). In the primary multivariate analysis (*n* = 567), head tilt was a significant risk factor for anisometropia (OR = 1.71, *p* = 0.013) and correlated with larger inter-eye differences in SE and axial length. In the sensitivity analysis (*n* = 258), the association between head tilt and anisometropia remained significant (OR = 1.94, *p* = 0.049) after adjusting for parental myopia and lifestyle factors. Although shoulder imbalance was initially associated with SE (*β* = −0.48, *p* = 0.030) and AL (*β* = 0.24, *p* = 0.025), this association became non-significant after including parental myopia and lifestyle-related factors. Forward head posture showed no significant associations with refractive parameters.

**Conclusion:**

Asymmetrical postural misalignments have distinct associations with refractive errors. Head tilt and poor writing posture exhibit distinct associations with anisometropia. These findings highlight the potential value of postural assessment in myopia management.

## 1 Introduction

Myopia has emerged as one of the leading causes of visual impairment worldwide, currently affecting nearly 2 billion people globally (1). It is estimated that nearly half of the world’s population will be myopic by 2050 (2), posing a growing public health concern due to the risk of sight-threatening complications. Myopia can be corrected optically by glasses, contact lenses, or refractive surgery. Nevertheless, it has been associated with complications, such as myopic macular degeneration (MMD), retinal detachment (RD), cataract, and open angle glaucoma (OAG). These complications can lead to irreversible visual impairment later in life (3). Anisometropia is regarded as an associated factor for myopia development, while myopia progression is a stimulus driving anisometropic development (4, 5). Anisometropia is a condition characterized by differing refractive errors in each eye, commonly defined by an interocular spherical equivalent difference of ≥1 diopter (D). Its prevalence increases with age: from approximately 7.8%–9.0% in children aged 7–9 years, to 16.0% by age 10, and reaching 39.0% by age 19 (6). In a study of Chinese children aged between 4 and 17 years, the mean prevalence of anisometropia was 13.2% (95% CI: 12.1%–14.2%) (7). Beyond its role as a primary cause of amblyopia, anisometropia induces binocular dysfunctions—including aniseikonia, strabismus and the disruption of fusion and stereopsis (8–10).

Therefore, a deeper understanding of myopia is essential for the effective prevention and control of the condition. The development of myopia is influenced by both genetic and environmental factors. Key risk factors include a family history of myopia, extended near-work duration, shorter reading distance, head tilt during writing, and insufficient outdoor time (1, 11, 12). In addition, higher baseline myopia, short near-working distances (<30 cm), and prolonged indoor activity such as reading and writing with insufficient outdoor exposure are risk factors for anisometropia (13, 14).

Poor postural habits during near work—such as forward head posture, head tilt, and holding reading material too close—are common among children and adolescents and have been linked not only to visual strain but also to musculoskeletal problems, including scoliosis. Previous studies have highlighted the relationship between body posture and the onset and progression of myopia, suggesting that continuous reading (>30 min) without breaks and shorter working distances (<30 cm) can significantly influence these conditions (15, 16). Children and adolescents with persistently incorrect posture had a high risk of myopia, which was 2.8 times higher than that in students who always maintained correct posture (17). Zhou et al. (6) found that children who screened positive for scoliosis are 4.08 times more likely to develop myopia compared with those without scoliosis. However, in most studies investigating poor posture among myopic children, assessments of postural anomalies rely mostly on questionnaires, which introduces inherent subjective influences.

Adolescence represents a critical phase of musculoskeletal and visual maturation. Early identification of postural abnormalities may play a crucial role in preventing or mitigating the onset and progression of myopia. The present study aims to evaluate the relationships between objective physical examination indicators, particularly postural anomalies such as head tilt, shoulder asymmetry, and forward head posture, and myopia as well as anisometropia among school-age children. By examining this relationship, the research seeks to clarify whether musculoskeletal development is associated with myopia and anisometropia, thereby expanding the understanding of its risk factors beyond traditional ophthalmologic frameworks. Unveiling these associations provides both a theoretical and empirical basis for the development of integrated screening, prevention, and intervention strategies, ultimately aiming to preserve and enhance visual function.

## 2 Methods

### 2.1 Study population

**Figure 1:**
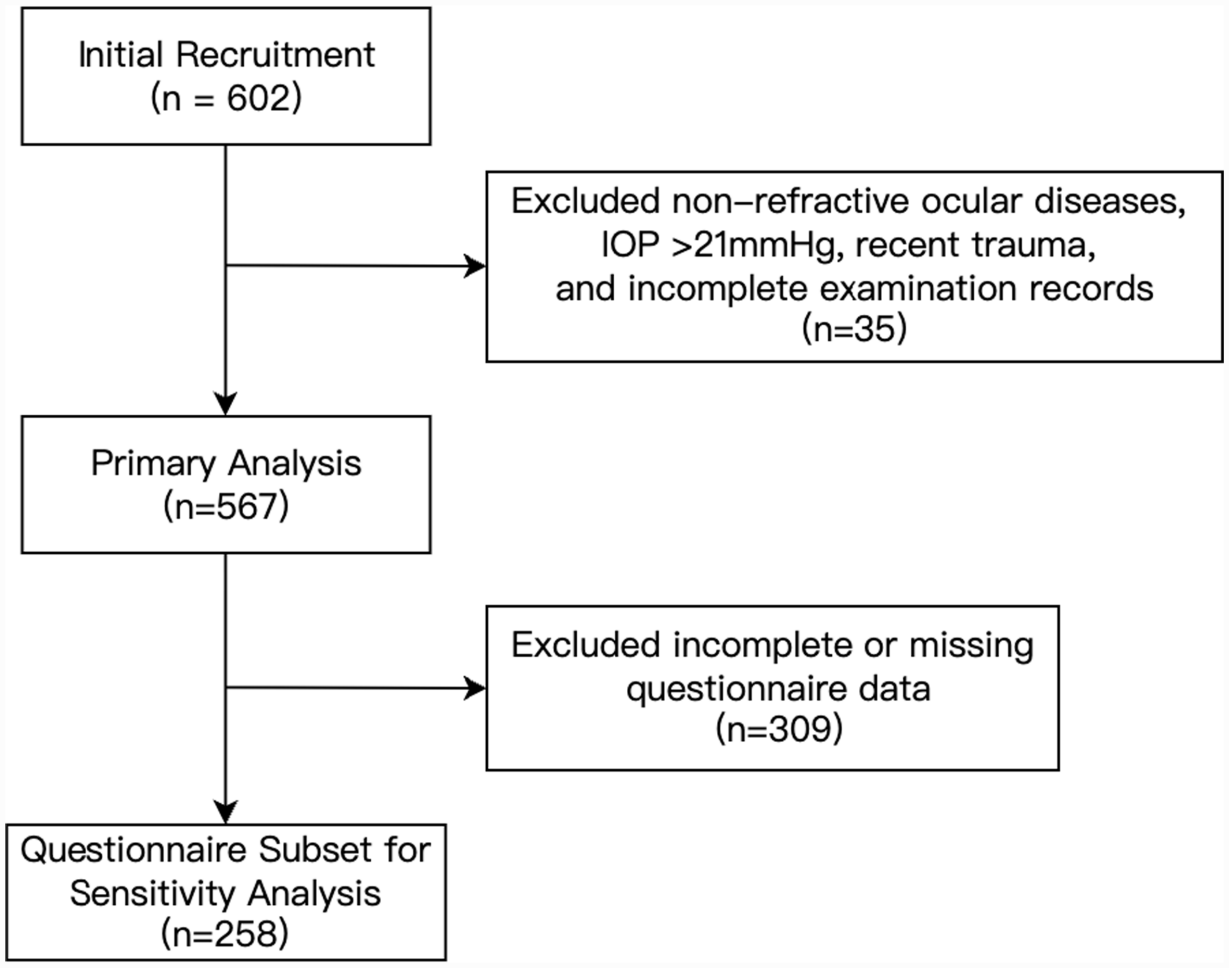
Flowchart for the inclusion and exclusion of the study participants.

A total of 602 students were initially recruited from one secondary school in Ziyang City, Sichuan Province, China. Inclusion criteria included students aged 12–18 years willing to participate in ocular and physical assessments. Exclusion criteria were rigorously applied to remove individuals with non-refractive ophthalmic diseases (e.g., amblyopia, glaucoma), recent trauma (within 6 months), abnormal IOP (>21 mmHg), manifest or latent strabismus, or impaired binocular function. Of the 602 students initially recruited, 35 were excluded based on these criteria, resulting in a final study population of 567 participants successfully included in the primary analysis. Among the 567 participants, parents of 258 participants completed a detailed questionnaire regarding myopia risk factors, including parental myopia history (none/one/both), daily sleep duration (hours), daily physical exercise frequency [low (<30 min), moderate (30 min–2 h), high (>2 h)], and self-reported poor writing posture frequency (rare, occasional, frequent). These participants constituted the questionnaire subset (*n* = 258), which was used for sensitivity analysis (Figure 1).

This study was approved by the Ethical Committee of the Sichuan Provincial People’s Hospital (No. 2024-720) and conducted in accordance with the guidelines of the Declaration of Helsinki.

### 2.2 Ocular and physical examinations

A comprehensive ophthalmic examination was conducted on all participants by professional optometrists, including measurements of distance visual acuity (VA) using logMAR, intraocular pressure (IOP) with a non-contact tonometer (Nidek NT-510), and detailed anterior segment evaluation via slit-lamp biomicroscopy. Ocular alignment and binocular function were assessed using near and distant cover/uncover tests, ocular motility evaluation. Axial length (AL) was precisely measured using laser interferometry (IOLMaster 500), taking the average of three consecutive readings. Cycloplegic refraction was determined after administering 1% cyclopentolate hydrochloride, utilizing an autorefractometer (Topcon KR-800), followed by a detailed fundus examination via Swept Source optical coherence tomography (OCT) (VG200S; SVision Imaging).

Postural assessment was performed using the Lantian Medical Postural Assessment Software LTM-ZS970 (Beijing Lantian Medical Equipment Co., LTD), a validated tool for the screening of postural abnormalities (18). The system’s photography module is equipped with an integrated Azure Kinect DK depth camera, which was positioned at a fixed height of 85 cm and a distance of 2.5 m from the floor markers. Prior to data collection, one trained researcher applied small circular adhesive markers to the earlobe and acromion to enhance the visibility and accuracy of landmark localization.

Participants were instructed to wear light clothing to ensure markers remained fully visible. Under stable lighting conditions against a clean background, participants stood on the floor markers and were instructed to look straight ahead at a target point positioned at eye level on a wall 5 m away, aligning their head and shoulders naturally. Photographs were captured from both frontal and lateral views. Three primary angles were analyzed: head tilt, shoulder imbalance, and forward head angle. To ensure accuracy, automatic landmark detection was followed by manual verification, and all examinations were conducted by the same trained professional to minimize inter-operator variability. To verify the consistency of landmark identification, repeated assessments were performed by two raters, yielding excellent reliability (intraclass correlation coefficient >0.90) for both intra- and inter-rater assessments.

### 2.3 Definitions of ocular outcomes and postural groups

Poor visual acuity was defined as the participant’s presenting visual acuity (i.e., with the participant’s habitual correction, if any) of either eye > 0.1 logMAR. Spherical equivalent (SE) was defined as the sum of the spherical refractive error plus half of the cylindrical (CYL) refractive error. Anisometropia was defined as the difference in cycloplegic inter-eye SE ≥1.00 D. The differences in inter-eye SE, and AL were presented in the form of absolute values. The SE of the right eye was used to define the refractive groups. Myopia was defined as SE ≤−0.50 D. Emmetropia was defined as −0.50 D < SE ≤ 0.50 D, and hyperopia was defined as SE > 0.50 D.

**Figure 2:**
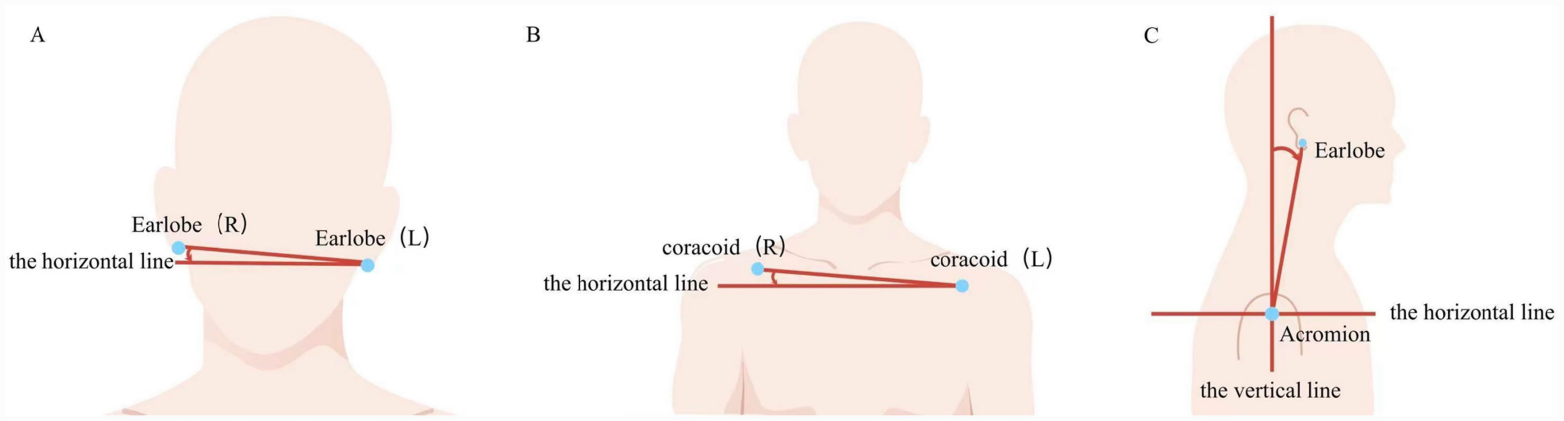
Illustration of postural assessment measurements. **(A)** Head tilt: Defined as the angle between the line connecting the earlobes and the horizontal line. The normal range is 0 ~ 3°; values exceeding 3° are considered abnormal. **(B)** Shoulder imbalance: determined by the deviation of the line connecting a line was drawn between the left and right coracoid process markers, also with a normal range of 0 ~ 1°, exceeding 1° are considered abnormal. **(C)** Head forward angle: Measured as the angle between the line connecting the earlobe and the acromion and the vertical line.

Head tilt (19) was defined as the degree of deviation of the line connecting the earlobes from the horizontal line, with a normal range of 0 ~ 3°; values exceeding 3° are considered abnormal based on established physiological symmetry norms (20) (Figure 2A). Shoulder imbalance was defined as the angle between the line connecting the bilateral acromion markers and the horizontal line, with a normal range of 0 ~ 1°, values >1° were considered abnormal (20) (Figure 2B). The head forward angle was measured as the angle between the line connecting the earlobe and acromion and the vertical line (Figure 2C), which serves as an exploratory indicator provided by the Lantian Medical system (18). Consistent with findings in previous head forward posture research (21), the measurements in our study followed a normal distribution. To facilitate analysis, head forward angle was categorized into three groups based on cut-off points defined by the sample mean (M) plus/minus one standard deviation (SD): Low Group: (0 ~ 4°); Medium Group: (5 ~ 15°); High Group:(16 ~ 31°), the Medium Group served as the reference group in subsequent regression analyses.

### 2.4 Statistical analysis

All analyses were conducted using SPSS 26.0 (IBM Corp, Armonk, NY, USA). Normally distributed continuous variables were expressed as mean ± standard deviation (Mean ± SD), while non-normally distributed variables were reported as median and interquartile range (IQR; P25, P75). Group comparisons employed independent *t*-tests/ANOVA (normally distributed) or Mann–Whitney U/Kruskal–Wallis H tests (non-normally distributed). Categorical variables were summarized as frequency and percentage [*n* (%)], with group differences assessed by chi-square tests (*χ*^2^).

Binary logistic regression and multiple linear regression models were constructed. Primary Analysis (overall sample, *n* = 567), models were adjusted for age, sex, height, weight, head tilt, shoulder imbalance, and head forward posture. Additionally, SE was included as a covariate in the anisometropia (yes/no) and inter-eye SE difference as outcomes, while AL was included as a covariate in models with inter-eye AL difference. Sensitivity analysis (questionnaire-completer sample, *n* = 258): models were additionally adjusted for questionnaire-derived covariates, including parental myopia status, sleep duration, physical exercise, and poor writing posture. Participants lacking relevant questionnaire information were completely excluded from this sensitivity analysis. Results are presented as adjusted odds ratios (OR) or regression coefficients (*β*) with 95% confidence intervals (95% CI). All tests were two-tailed, with statistical significance defined at *α* = 0.05.

## 3 Results

### 3.1 Characteristics of the study population in overall sample (*n* = 567) and questionnaire-completer sample (*n* = 258)

As detailed in Table 1, the study included 567 participants ranging in age from 12 to 18 years (mean age: 14.96 ± 1.60 years; 43.4% male). The prevalence of myopia was 82.5%, while emmetropia and hyperopia were present in 13.4% and 4.1% of the population, respectively. Poor visual acuity was observed in 44.3% of the total sample. The mean spherical equivalent (SE) of the right eye was −2.65 ± 2.13 D, the mean axial length (AL) was 24.71 ± 1.06 mm. The prevalence of head tilt and shoulder imbalance in the full sample was 20.3% (*n* = 115) and 20.1% (*n* = 114), respectively. For forward head posture, participants were categorized into low (17.6%, *n* = 100), medium (64.6%, *n* = 366), and high (17.8%, *n* = 101) groups.

**Table 1:** Baseline demographic and clinical characteristics of full sample (*n* = 567) and myopia risk factors in the questionnaire subset (*n* = 258).

Associated factors	Total	Non-anisometropia	Anisometropia	*p*
Age, years	14.96 ± 1.60	14.99 ± 1.57	14.90 ± 1.67	0.533
Sex, male, *n* (%)	246 (43.4%)	154 (41.6%)	92 (46.7%)	0.245
Height, cm	161.74 ± 7.82	161.23 ± 7.75	162.69 ± 7.88	0.034*
Weight, kg	56.31 ± 12.96	55.91 ± 12.37	57.07 ± 14.00	0.312
Head tilt, *n* (%)	115 (20.3%)	64 (17.3%)	51 (25.9%)	0.015*
Shoulder imbalance, *n* (%)	114 (20.1%)	78 (21.1%)	36 (18.3%)	0.427
Head forward posture				0.905
Low group, *n* (%)	100 (17.6%)	66 (17.8%)	34 (17.3%)	
Medium group, *n* (%)	366 (64.6%)	240 (64.9%)	126 (64.0%)	
High Group, *n* (%)	101 (17.8%)	64 (17.3%)	37 (18.8%)	
Poor visual acuity, *n* (%)	251 (44.3%)	132 (35.7%)	119 (60.4%)	<0.001*
Ametropia Status, *n* (%)				0.051
Myopia	468 (82.5%)	296 (80.0%)	172 (87.3%)	
Emmetropia	76 (13.4%)	59 (16.0%)	17 (8.6%)	
Hyperopia	23 (4.1%)	15 (4.1%)	8 (4.1%)	
SE, D	−2.65 ± 2.13	−2.64 ± 2.20	−2.68 ± 2.00	0.830
AL, mm	24.71 ± 1.06	24.71 ± 1.10	24.70 ± 0.99	0.898
Difference in inter-eye SE, D	0.63 (0.25, 1.25)	0.37 (0.13, 0.62)	1.63 (1.25, 2.50)	<0.001*
Difference in inter-eye AL, mm	0.24 (0.11, 0.50)	0.15 (0.07, 0.24)	0.66 (0.42, 1.05)	<0.001*
Parental myopia, *n* (%)^a^				0.677
None	130 (50.4%)	89 (51.4%)	41 (48.2%)	
Either one	100 (38.8%)	64 (37.0%)	36 (42.4%)	
Both	28 (10.9%)	20 (11.6%)	8 (9.4%)	
Sleep time, hours^a^	7.72 ± 0.96	7.66 ± 0.98	7.83 ± 0.92	0.181
Frequency of daily physical exercise, *n* (%)^a^				0.820
Low (<30 min/day)	13 (5.0%)	8 (4.6%)	5 (5.9%)	
Moderate (30 min–2 h/day)	106 (41.1%)	73 (42.2%)	33 (38.8%)	
High (>2 h/day)	139 (53.9%)	92 (53.2%)	47 (55.3%)	
Poor writing posture, *n* (%)^a^				0.092
Rare/None	95 (36.8%)	67 (38.7%)	28 (32.9%)	
Occasional	105 (40.7%)	74 (42.8%)	31 (36.5%)	
Frequent	58 (22.5%)	32 (18.5%)	26 (30.6%)	

SE, spherical equivalent; AL, axial length. *Indicates statistical significance (*p* < 0.05).

a Data for these variables were available only for participants whose parents completed the questionnaire (*n* = 258).

Comparison between the questionnaire subset (*n* = 258), the excluded sample (*n* = 309), and the full sample (*n* = 567) revealed that, except for a slight difference in age (14.52 ± 1.52 vs. 15.32 ± 1.57 vs. 14.96 ± 1.60 years, respectively; *p <* 0.001), no significant differences existed in other baseline characteristics, supporting the representativeness of the questionnaire subset (Supplementary Table S1).

### 3.2 Association between postural misalignment and myopia severity

As shown in Supplementary Table S2, in the primary multivariate analysis (*n* = 567), older age (*β* = −0.11, *p* = 0.048), female sex (*β* = −0.54, *p =* 0.018), and the presence of shoulder imbalance (*β* = −0.48, *p =* 0.030) were significant predictors of a more myopic SE. For AL, greater height (*β* = 0.03, *p <* 0.001) and shoulder imbalance (*β* = 0.24, *p =* 0.025) were independently associated with longer AL. In the sensitivity analysis (*n* = 258), the association between shoulder imbalance and SE/AL became non-significant (*p* = 0.227, *p* = 0.243) after adjusting for parental myopia and lifestyle factors. Parental myopia emerged as the dominant predictor, compared to no parental myopia, having one or both myopic parents was associated with a significantly more myopic SE (one: *β* = −1.33, *p <* 0.001; both: *β* = −1.39, *p =* 0.002) and longer AL (one: *β* = 0.48, *p =* 0.001; both: *β* = 0.43, *p =* 0.046). No significant associations were found between head tilt or other forward head posture categories and mean SE or AL in any of the adjusted models.

### 3.3 Association between postural misalignment and anisometropia, interocular differences in SE and AL

As shown in Table 1, Participants were categorized into non-anisometropia (*n* = 370) and anisometropia (*n* = 197, 34.7%) groups. Participants with anisometropia were significantly taller than those without (162.69 ± 7.88 cm vs. 161.23 ± 7.75; *p =* 0.034). Regarding postural alignment, a significantly higher prevalence of head tilt was observed in the anisometropia group (25.9% vs. 17.3%; *p =* 0.015). Other postural factors, such as shoulder imbalance and forward head posture, did not show statistically significant differences (all *p >* 0.05). Additionally, poor visual acuity was markedly more prevalent in the anisometropia group (60.4% vs. 35.7%; *p <* 0.001). In the anisometropia group, the median inter-eye differences for SE and AL were 1.63 D (IQR, 1.25–2.50 D) and 0.66 mm (IQR, 0.42–1.05 mm), respectively.

In the primary multivariate logistic regression (*n* = 567), head tilt was identified as a significant independent predictor of anisometropia (OR = 1.71, 95% CI: 1.12–2.61; *p =* 0.013). Furthermore, linear regression confirmed that head tilt was positively associated with the magnitude of inter-eye SE difference (*β* = 0.22, 95% CI: 0.03–0.42; *p =* 0.026) and AL difference (*β* = 0.11, 95% CI: 0.03–0.20; *p =* 0.007) (Table 2).

**Table 2:** Multivariate regression analyses of postural misalignment associated with anisometropia and inter-eye differences.

Associated factors	Anisometropia				Difference in inter-eye SE				Difference in inter-eye AL			
	Primary analysis^a^		Sensitivity analysis^b^		Primary analysis^a^		Sensitivity analysis^b^		Primary analysis^a^		Sensitivity analysis^b^	
	OR (95% CI)	*p*	OR (95% CI)	*p*	β (95% CI)	*p*	β (95% CI)	*p*	β (95% CI)	*p*	β (95% CI)	*p*
Head tilt (ref. = no)	1.71 (1.12–2.61)	0.013*	1.94 (1.00–3.77)	0.049*	0.22 (0.03–0.42)	0.026*	0.14 (−0.13–0.42)	0.309	0.11 (0.03–0.20)	0.007*	0.09 (−0.03–0.21)	0.164
Shoulder imbalance (ref. = no)	0.79 (0.50–1.24)	0.302	0.72 (0.36–1.45)	0.361	−0.04 (−0.24–0.15)	0.672	0.00 (−0.27–0.28)	0.974	−0.03 (−0.11–0.06)	0.537	0.00 (−0.12–0.13)	0.946
Head forward (ref. = medium group)												
Low group	0.96 (0.60–1.55)	0.876	1.47 (0.73–2.96)	0.276	0.03 (−0.18–0.24)	0.786	0.15 (−0.14–0.44)	0.321	0.00 (−0.09–0.09)	0.973	0.05 (−0.08–0.18)	0.431
High group	0.96 (0.60–1.56)	0.880	0.82 (0.37–1.83)	0.627	−0.02 (−0.24–0.19)	0.836	0.01 (−0.31–0.32)	0.975	−0.04 (−0.13–0.05)	0.415	−0.02 (−0.17–0.12)	0.741

a (Primary analysis, *n* = 567): Multivariate model for the full sample, adjusted for Age, Sex, Height, Weight, Head tilt, Shoulder imbalance, Head forward posture and SE (for Anisometropia and SE difference models) or AL (for AL difference model).

b (Sensitivity analysis, *n* = 258): Multivariate model for the questionnaire subset, adjusted for Age, Sex, Height, Weight, Head tilt, Shoulder imbalance, Head forward posture, SE (for Anisometropia and SE difference) or AL (for AL difference), Parental myopia history, Sleep duration, Physical exercise, and Poor writing posture.

SE, spherical equivalent; AL, axial length; *Indicates statistical significance (*p* < 0.05).

In the sensitivity analysis (*n* = 258), the association between head tilt and the presence of anisometropia remained significant (OR = 1.94, 95% CI: 1.00–3.77; *p =* 0.049), but it was no longer significantly associated with the magnitude of inter-eye differences in SE (*p =* 0.309) and AL (*p =* 0.164). Shoulder imbalance and forward head posture showed no significant associations with anisometropia or inter-eye differences in any of the models (Table 2). Additionally, frequent poor writing posture was identified as an independent risk factor for anisometropia (OR = 2.53, 95% CI: 1.14–5.63; *p* = 0.022) In contrast, other factors—including demographics, SE and AL, and other questionnaire-related variables—showed no significant associations in the fully adjusted models (Supplementary Table S3).

### 3.4 Demographic associations with postural misalignment

The general demographic characteristics across the three postural misalignment categories are presented in Supplementary Table S4. Shoulder imbalance was significantly associated with greater height (163.34 ± 8.07 cm vs. 161.33 ± 7.72 cm; *p =* 0.014), while the high forward head posture group had a higher proportion of male participants [57.4% vs. 46.0% (Low) and 38.8% (Medium) ; *p* = 0.003], greater height [165.08 ± 8.21 cm vs. 160.92 ± 7.42 cm (Low) and 161.34 ± 8.05 cm (Medium) *p* < 0.001], and higher weight [59.85 ± 14.90 kg vs. 55.68 ± 12.33 kg (Low) and 55.04 ± 12.67 kg (Medium) p = 0.009]. No significant demographic associations were observed for head tilt (all *p >* 0.05).

## 4 Discussion

This study provides objective, photographic evidence of a significant association between specific postural misalignments and ocular parameters in secondary school students, a demographic characterized by heavy academic workloads and a high prevalence of refractive errors (2, 22). The principal finding is that anisometropia prevalence was significantly elevated among students exhibiting head tilt compared to those with a neutral posture. The association between head tilt and anisometropia remained significant (*p* < 0.05) in both primary and sensitivity analyses. Notably, the OR increased from 1.71 to 1.94 after adjusting for additional confounders, underscoring head tilt as an independent risk factor for anisometropia.

Our examinations, while conducted in a standardized standing position, effectively capture chronic postural abnormalities. It is established that head tilt can alter binocular alignment and impair vision. Zhou et al. (6) found that the prevalence of anisometropia was 4.08 times higher in students with scoliosis. Similarly, Li et al. (23) identified a significant association between head turning during writing and greater myopia, as well as longer axial length. Our study, however, did not find a similar association. This discrepancy likely stems from two methodological differences. First, Li’s study relied on subjective parent questionnaires, whereas our study used objective photographic measurements. Second, Li assessed head posture during seated activities like writing, while we measured it in a standing position. These differences in measurement technique and body posture limit the direct comparability of the results.

One of the primary mechanisms likely underlying this association is the creation of an aniso-accommodative demand. A persistent head tilt during near work may lead to different viewing distances and angles for each eye, forcing them to accommodate or focus unequally. This asymmetric visual demand, especially over long periods, may lead to different hyperopic defocus signals in each retina, the dominant eye displays a smaller accommodative lag (*p* < 0.001) (24), resulting in the differential of axial elongation between the eyes (10, 23). This unequal growth stimulus could plausibly drive the asymmetric development of the eyes, resulting in anisometropia.

In our primary multivariate analysis (*n* = 567), head tilt was significantly associated with a greater magnitude of inter-eye differences in both spherical equivalent (SE) and axial length (AL). However, in the sensitivity analysis (*n* = 258), after further adjustment for parental myopia and lifestyle factors, the association with the magnitude of these differences did not retain statistical significance (SE: *p* = 0.309; AL: *p* = 0.164). This may be attributed to the limited sample size within the sensitivity analysis or a potential threshold effect, suggesting that the association may only manifest when the severity of misalignment exceeds a critical level. Consequently, future investigations with larger cohorts are warranted to explore this relationship further.

In the primary analysis, shoulder imbalance correlated with higher myopia and longer AL. However, this association lost significance in the fully adjusted sensitivity analysis. This loss of significance likely suggests that shoulder imbalance is a functional manifestation of cumulative lifestyle habits—physical stresses (25), rather than an independent driver of refractive change. Besides, the reduced sample size in the questionnaire subset (*n* = 258) may have further limited the statistical power. Conversely, parental myopia status exerted a pronounced influence on both SE and AL. Consistent with previous research findings (26, 27), Multivariate analysis revealed that compared to students with no myopic parents, those with either one or both myopic parents exhibited significantly greater myopic refractive errors.

Moreover, parent-reported poor writing posture was identified as an independent indicator of anisometropia (OR = 2.53, 95% CI: 1.14–5.63). A similar finding was reported in another study; specifically, near work performed at a distance of less than 30 cm (OR = 1.33, 95% CI: 1.08–1.64) was significantly associated with an increased risk of incident anisometropia (14). These findings underscore the importance for parents and teachers to pay attention to the timely correction of students’ poor writing posture.

In contrast, forward head posture showed no significant association with ocular parameters in primary analysis and sensitivity analysis. Several factors may explain this observation. First, as we noted, our measurement method using the earlobe and acromion differs from the more standard Craniovertebral Angle (CVA)– defined as the angle between the vertebral prominence of C7, the tragus, and a horizontal line (28), which may capture different aspects of cervical posture. Second, the static standing posture we measured may not accurately reflect the dynamic, flexed posture adopted during prolonged near work. Students may maintain a relatively neutral posture when standing but adopt significant forward head flexion for hours while studying, and it is this dynamic posture that would exert influence on the visual system. Besides, severe forward head posture (high group) is more prevalent among taller and heavier male students. These results show postural parameters are significantly influenced by demographic factors. This underscores the need for future large-scale studies to establish age-specific diagnostic thresholds for adolescents. Unlike questionnaire-based studies, our research objectively assessed postural abnormalities of the head and shoulders in a standing position using photography, providing insights into chronic postural issues. This objective and accessible method suggests that basic postural screening could be a valuable addition to routine pediatric and school-based health examinations. Identifying students with significant asymmetries like head tilt could flag them as being at higher risk for anisometropia. This supports a new avenue for anisometropia control, where ergonomic advice and postural interventions could complement traditional strategies.

In our study, the prevalence of anisometropia was 34.74%, which is higher than in previous studies (7, 13), likely reflecting our voluntary recruitment design and the high myopia rate (82.54%) of the sample. The rate of poor visual acuity was also higher than the 25.75% reported in Hong Kong (29). This discrepancy may be due to regional differences in access to professional eye care and socioeconomic factors influencing the frequency of eye examinations. A prior study in Chengdu also reported a 31.1% under-correction rate among children (30), which closely mirrors our observations and suggests a persistent issue of suboptimal refractive correction among school-aged children in this region.

Several limitations of this study should be noted. First, the study’s cross-sectional design, combined with voluntary recruitment from a single secondary school, inherently limits the interpretation and generalizability of the findings. The cross-sectional nature precludes causal inference between posture and ocular parameters, as well as the potential for selection bias. Second, regarding physical measurements, while we standardized procedures to minimize variability, shoulder position may still influence the assessment of forward head angle. Additionally, the stratification of forward head posture relied on the study group’s statistical distribution (Mean ± SD) due to the absence of established clinical cutoffs; this limits external comparability and underscores the necessity for standardized diagnostic criteria. An additional limitation is that the questionnaire-based sensitivity analysis may be subject to volunteer bias and we could not control for all lifestyle confounders, such as the specific duration of smartphone use, daily near-work hours, etc., which may involve different postural demands.

To build upon our findings, mutli-site and longitudinal studies are essential to track students’ postural and ocular development over several years to better understand causality. Furthermore, studies utilizing dynamic motion capture to analyze posture during near-work tasks would provide more direct evidence of the biomechanical stresses placed on the visual system. Finally, interventional trials are needed to determine if correcting postural misalignments through physiotherapy, ergonomic adjustments, or targeted exercises can slow the progression of anisometropia.

In conclusion, our study provides objective evidence that postural misalignments are significantly associated with refractive errors in adolescents. Specifically, the prevalence of anisometropia was significantly elevated among students exhibiting head tilt compared to those with a neutral posture. Abnormal posture may be associated with misalignment of the binocular position, which could contribute to interocular differences in refractive status. These findings suggest a potential link between musculoskeletal alignment and visual development during a critical developmental period. Therefore, encouraging students to correct their postures and maintain proper reading and writing habits may help reduce the prevalence of anisometropia and promote better binocular vision.

## Data Availability

The raw data supporting the conclusions of this article will be made available by the authors, without undue reservation.
