# Supplementary material for: Associations of postural asymmetry with refractive error: an objective photogrammetric analysis in students
Source: Front Med (Lausanne). 2026 Jan 29;13:1727688. doi: 10.3389/fmed.2026.1727688 (PMC12893978; doi:10.3389/fmed.2026.1727688)
Supplement: Supplementary file 1 [file Table_1.DOCX]

Supplementary Material

# Supplementary Table

Supplementary Table 1: Comparison of baseline characteristics between the Questionnaire subset (n=258), Excluded sample (n=309) and the Full sample (n=567)

| **Associated factors** | **Questionnaire subset** | **Excluded sample** | **Full sample** | **P** |
| --- | --- | --- | --- | --- |
| **Age, years** | 14.52 ± 1.52 | 15.32 ± 1.57 | 14.96 ± 1.60 | **< 0.001*** |
| **Sex, male, n (%)** | 112 (43.4%) | 134 (43.4%) | 246 (43.4%) | 1.000 |
| **Height, cm** | 161.36 ± 7.23 | 162.05 ± 8.29 | 161.74 ± 7.82 | 0.581 |
| **Weight, kg** | 56.09 ± 13.27 | 56.50 ± 12.72 | 56.31 ± 12.96 | 0.932 |
| **Head tilt, n (%)** | 55 (21.3%) | 60 (19.4%) | 115 (20.3%) | 0.855 |
| **Shoulder Imbalance, n (%)** | 54 (20.9%) | 60 (19.4%) | 114 (20.1%) | 0.905 |
| **Head Forward Posture, n (%)** |  |  |  | 0.880 |
| Low Group | 50 (19.4%) | 50 (16.2%) | 100 (17.6%) |  |
| Medium Group | 165 (64.0%) | 201 (65.0%) | 366 (64.6%) |  |
| High Group | 43 (16.7%) | 58 (18.8%) | 101 (17.8%) |  |
| **Poor visual acuity, n (%)** | 109 (42.2%) | 142 (46.0%) | 251 (44.3%) | 0.676 |
| **Ametropia, n (%)** |  |  |  | 0.882 |
| Myopia | 211 (81.8%) | 257 (83.2%) | 468 (82.5%) |  |
| Emmetropia | 34 (13.2%) | 42 (13.6%) | 76 (13.4%) |  |
| Hyperopia | 13 (5.0%) | 10 (3.2%) | 23 (4.1%) |  |
| **SE, D** | -2.63 ± 2.19 | -2.67 ± 2.08 | -2.65 ± 2.13 | 0.971 |
| **AL, mm** | 24.74 ± 1.05 | 24.68 ± 1.06 | 24.71 ± 1.06 | 0.767 |
| **Anisometropia, n (%)** | 85 (32.9%) | 112 (36.2%) | 197 (34.7%) | 0.713 |
| **Difference in inter-eye SE, D** | 0.62 (0.25, 1.25) | 0.63 (0.25, 1.25) | 0.63 (0.25, 1.25) | 0.894 |
| **Difference in inter-eye AL, mm** | 0.24 (0.10, 0.47) | 0.24 (0.12, 0.54) | 0.24 (0.11, 0.50) | 0.703 |

SE: Spherical Equivalent; AL: Axial Length. Normally distributed variables are expressed as mean ± SD and compared using one-way ANOVA. Categorical variables are expressed as n (%) and compared using Pearson’s χ test. Non-normally distributed variables are expressed as median (P25, p75) and compared using the Kruskal-Wallis H test. ^*^ Indicates statistical significance (P < 0.05).

Supplementary Table 2: Multivariate regression analyses of factors associated with SE and AL

| **Associated factors** | **Spherical Equivalent (SE, D)** | | | | **Axial Length (AL, mm)** | | | |
| --- | --- | --- | --- | --- | --- | --- | --- | --- |
|  | **Primary Analysis^a^** | | **Sensitivity Analysis^b^** | | **Primary Analysis^a^** | | **Sensitivity Analysis^b^** | |
|  | **β (95% CI)** | **P** | **β (95% CI)** | **P** | **β (95% CI)** | **P** | **β (95% CI)** | **P** |
| **Age, years** | -0.11 (-0.23, 0.00) | **0.048*** | -0.20 (-0.39, -0.02) | **0.031*** | 0.01 (-0.04, 0.07) | 0.653 | 0.09 (0.01, 0.18) | **0.036*** |
| **Sex (ref.=male)** | -0.54 (-0.99, -0.09) | **0.018*** | -0.55 (-1.22, 0.12) | 0.111 | -0.16 (-0.38, 0.06) | 0.144 | -0.16 (-0.47, 0.16) | 0.333 |
| **Height, cm** | -0.02 (-0.05, 0.02) | 0.349 | -0.01 (-0.06, 0.04) | 0.596 | 0.03 (0.01, 0.05) | **<0.001*** | 0.03 (0.01, 0.06) | **0.005*** |
| **Weight, kg** | 0.01 (0.00, 0.03) | 0.114 | 0.01 (-0.01, 0.03) | 0.429 | 0.00 (-0.01, 0.00) | 0.361 | -0.01 (-0.02, 0.00) | 0.250 |
| **Head tilt (ref.=No)** | 0.06 (-0.37, 0.50) | 0.780 | -0.23 (-0.88, 0.41) | 0.482 | -0.05 (-0.26, 0.16) | 0.663 | 0.12 (-0.18, 0.43) | 0.427 |
| **Shoulder Imbalance (ref.=No)** | -0.48 (-0.92, -0.05) | **0.030*** | -0.40 (-1.04, 0.25) | 0.227 | 0.24 (0.03, 0.45) | **0.025*** | 0.18 (-0.12, 0.48) | 0.243 |
| **Head Forward (ref.=Medium Group)** |  |  |  |  |  |  |  |  |
| Low Group | -0.12 (-0.59, 0.34) | 0.601 | 0.02 (-0.65, 0.70) | 0.947 | -0.04 (-0.26, 0.19) | 0.755 | -0.09 (-0.40, 0.23) | 0.598 |
| High Group | 0.19 (-0.29, 0.67) | 0.440 | 0.17 (-0.58, 0.92) | 0.440 | -0.23 (-0.46, 0.00) | 0.050 | -0.36 (-0.71, -0.01) | 0.050 |
| **Parental myopia (ref.=None)** |  |  |  |  |  |  |  |  |
| Either one myopic | -- | -- | -1.33 (-1.89, -0.76) | **<0.001*** | -- | -- | 0.48 (0.21, 0.74) | **0.001*** |
| Both myopic | -- | -- | -1.39 (-2.27, -0.51) | **0.002*** | -- | -- | 0.43 (0.01, 0.84) | **0.046*** |
| **Sleep duration (hour)** | -- | -- | -0.14 (-0.43, 0.14) | 0.327 | -- | -- | 0.08 (-0.05, 0.22) | 0.242 |
| **Physical exercise (ref.=High [>2 hours/day])** |  |  |  |  |  |  |  |  |
| Low (<30 min/day) | -- | -- | -0.06 (-0.66, 0.55) | 0.859 | -- | -- | -0.02 (-0.30, 0.27) | 0.911 |
| Moderate (30 min–2 h/day) | -- | -- | 0.23 (-1.03, 1.50) | 0.716 | -- | -- | -0.22 (-0.82, 0.37) | 0.465 |
| **Poor Writing posture (ref.=Rare/None)** |  |  |  |  |  |  |  |  |
| Occasional | -- | -- | 0.45 (-0.20, 1.10) | 0.175 | -- | -- | -0.15 (-0.46, 0.15) | 0.323 |
| Frequent | -- | -- | -0.13 (-0.89, 0.63) | 0.740 | -- | -- | 0.15 (-0.21, 0.51) | 0.420 |

**^a^** Primary Analysis, n=567: Multivariate linear regression model adjusted for Age, Sex, Height, Weight, Head tilt, Shoulder imbalance, and Head forward posture.

**^b^** Sensitivity analysis, n=258: Multivariate linear regression model adjusted for Age, Sex, Height, Weight, Head tilt, Shoulder imbalance, Head forward posture, Parental myopia history, Sleep duration, Physical exercise, and Poor writing posture.

SE: Spherical Equivalent; AL: Axial Length; ^*^ Indicates statistical significance (P**<**0.05).

Supplementary Table 3: Multivariate regression analyses of factors associated with anisometropia and inter-eye differences

| Associated Factors | Anisometropia | | | | Difference in inter-eye SE | | | | Difference in inter-eye AL | | | |
| --- | --- | --- | --- | --- | --- | --- | --- | --- | --- | --- | --- | --- |
|  | Primary Analysis^a^ | | Sensitivity Analysis^b^ | | Primary Analysis^a^ | | Sensitivity Analysis^b^ | | Primary Analysis^a^ | | Sensitivity Analysis^b^ | |
|  | OR (95% CI) | P | OR (95% CI) | P | β (95% CI) | P | β (95% CI) | P | β (95% CI) | P | β (95% CI) | P |
| **Demographics** |  |  |  |  |  |  |  |  |  |  |  |  |
| Age, years | 0.94 (0.84–1.06) | 0.310 | 0.88 (0.72–1.08) | 0.217 | -0.02 (-0.08–0.03) | 0.338 | -0.04 (-0.12–0.04) | 0.327 | -0.01 (-0.03–0.01) | 0.438 | -0.02 (-0.05–0.02) | 0.392 |
| Sex (ref.=male) | 1.01 (0.64–1.59) | 0.957 | 0.58 (0.29–1.18) | 0.136 | 0.10 (-0.10–0.31) | 0.319 | -0.06 (-0.35–0.23) | 0.679 | 0.06 (-0.03–0.15) | 0.167 | -0.02 (-0.15–0.10) | 0.722 |
| Height, cm | 1.03 (1.00–1.07) | 0.058 | 0.97 (0.92–1.02) | 0.284 | 0.01 (0.00–0.02) | 0.178 | -0.01 (-0.03–0.01) | 0.442 | 0.01 (0.00–0.01) | 0.114 | 0.00 (-0.01–0.01) | 0.433 |
| Weight, kg | 1.00 (0.98–1.01) | 0.863 | 1.01 (0.98–1.03) | 0.508 | 0.01 (0.00–0.01) | 0.172 | 0.01 (0.00–0.02) | 0.156 | 0.00 (0.00–0.00) | 0.275 | 0.00 (0.00–0.01) | 0.242 |
| **Ocular Covariates** |  |  |  |  |  |  |  |  |  |  |  |  |
| Spherical Equivalent (SE) | 0.98 (0.90–1.06) | 0.615 | 0.99 (0.86–1.12) | 0.831 | -0.02 (-0.06–0.02) | 0.241 | -0.02 (-0.07–0.04) | 0.564 | -- | -- | -- | -- |
| Axial Length (AL) | -- | -- | -- | -- | -- | -- | -- | -- | 0.03 (0.00–0.06) | 0.063 | 0.04 (-0.01–0.09) | 0.132 |
| **Genetic & Lifestyle Factors** |  |  |  |  |  |  |  |  |  |  |  |  |
| **Parental myopia (ref.=None)** |  |  |  |  |  |  |  |  |  |  |  |  |
| Either one myopic | -- | -- | 1.26 (0.68–2.35) | 0.464 | -- | -- | 0.00 (-0.25–0.25) | 0.997 | -- | -- | 0.01 (-0.10–0.12) | 0.862 |
| Both myopic | -- | -- | 0.80 (0.30–2.14) | 0.658 | -- | -- | -0.09 (-0.48–0.29) | 0.631 | -- | -- | -0.03 (-0.20–0.13) | 0.702 |
| Sleep duration (per hour) | -- | -- | 1.25 (0.92–1.70) | 0.146 | -- | -- | 0.05 (-0.08–0.17) | 0.454 | -- | -- | 0.01 (-0.04–0.07) | 0.619 |
| **Physical exercise (ref.=High [>2 hours/day])** |  |  |  |  |  |  |  |  |  |  |  |  |
| Low (<30 min/day) | -- | -- | 0.82 (0.43–1.55) | 0.534 | -- | -- | 0.04 (-0.22–0.30) | 0.751 | -- | -- | 0.03 (-0.09–0.14) | 0.634 |
| Moderate (30 min–2 h/day) | -- | -- | 0.58 (0.16–2.16) | 0.419 | -- | -- | -0.16 (-0.70–0.38) | 0.572 | -- | -- | -0.07 (-0.31–0.17) | 0.562 |
| **Poor Writing posture (ref.=Rare/None)** |  |  |  |  |  |  |  |  |  |  |  |  |
| Occasional | -- | -- | 1.09 (0.54–2.19) | 0.804 | -- | -- | -0.22 (-0.50–0.06) | 0.125 | -- | -- | -0.09 (-0.21–0.03) | 0.150 |
| Frequent | -- | -- | **2.53 (1.14–5.63)** | **0.022*** | -- | -- | 0.27 (-0.06–0.59) | 0.110 | -- | -- | 0.07 (-0.08–0.21) | 0.366 |

^a^ Primary Analysis, n=567: Multivariate model adjusted for Age, Sex, Height, Weight, Head tilt, Shoulder imbalance, Head forward, and SE (for Anisometropia/ Difference in inter-eye SE models) or AL (for Difference in inter-eye AL).

^b^ Sensitivity analysis, n=258: Multivariate model adjusted for Age, Sex, Height, Weight, Head tilt, Shoulder imbalance, Head forward posture, SE (for Anisometropia and Difference in inter-eye SE models) or AL (for Difference in inter-eye AL), Parental myopia history, Sleep duration, Physical exercise, and Poor writing posture.

SE: Spherical Equivalent; AL: Axial Length; ^*^ Indicates statistical significance (P**<**0.05).

Supplementary Table 4: Demographic characteristics of participants according to postural status

| **Associated factors** | **Head Tilt** | | **P** | **Shoulder Imbalance** | | **P** | **Head Forward Posture** | | | **P** |
| --- | --- | --- | --- | --- | --- | --- | --- | --- | --- | --- |
|  | **No (n=452)** | **Yes (n=115)** |  | **No (n=453)** | **Yes (n=114)** |  | **Low Group (n=100)** | **Medium Group (n=366)** | **High Group (n=101)** |  |
| **Age (years)** | 14.98±1.59 | 14.84±1.66 | 0.399 | 14.92±1.60 | 15.11±1.61 | 0.238 | 15.04±1.58 | 14.94±1.72 | 14.67±1.52 | 0.127 |
| **Sex (male, n (%)** | 204 (45.1%) | 42 (36.5%) | 0.096 | 188 (41.5%) | 58 (50.9%) | 0.071 | 46 (46.0%) | 142 (38.8%) | 58 (57.43%) | **0.003**^*^ |
| **Height (cm)** | 161.89±7.78 | 161.11±8.01 | 0.340 | 161.33±7.72 | 163.34±8.07 | **0.014**^*^ | 160.92±7.42 | 161.34±8.05 | 165.08±8.21 | **< 0.001**^*^ |
| **Weight (kg)** | 56.11±13.08 | 57.10±12.51 | 0.465 | 56.01±12.69 | 57.51±13.97 | 0.269 | 55.68±12.33 | 55.04±12.67 | 59.85±14.90 | **0.009**^*^ |

^*^ Indicates statistical significance (P**<**0.05).
